# Predicting the potential associations between circRNA and drug sensitivity using a multisource feature‐based approach

**DOI:** 10.1111/jcmm.18591

**Published:** 2024-09-30

**Authors:** Shuaidong Yin, Peng Xu, Yefeng Jiang, Xin Yang, Ye Lin, Manyu Zheng, Jinpeng Hu, Qi Zhao

**Affiliations:** ^1^ School of Computer Science and Software Engineering University of Science and Technology Liaoning Anshan China; ^2^ School of Electronic and Information Engineering University of Science and Technology Liaoning Anshan China; ^3^ College of Computer Science and Technology Jilin University Changchun China

**Keywords:** circRNA, drug, graph neural network, non‐negative matrix factorization, sparse autoencoder

## Abstract

The unique non‐coding RNA molecule known as circular RNA (circRNA) is distinguished from conventional linear RNA by having a longer half‐life, greater degree of conservation and inherent solidity. Extensive research has demonstrated the profound impact of circRNA expression on cellular drug sensitivity and therapeutic efficacy. There is an immediate need for the creation of efficient computational techniques to anticipate the potential correlations between circRNA and drug sensitivity, as classical biological research approaches are time‐consuming and costly. In this work, we introduce a novel deep learning model called SNMGCDA, which aims to forecast the relationships between circRNA and drug sensitivity. SNMGCDA incorporates a diverse range of similarity networks, enabling the derivation of feature vectors for circRNAs and drugs using three distinct calculation methods. First, we utilize a sparse autoencoder for the extraction of drug characteristics. Subsequently, the application of non‐negative matrix factorization (NMF) enables the identification of relationships between circRNAs and drugs based on their shared features. Additionally, the multi‐head graph attention network is employed to capture the characteristics of circRNAs. After acquiring the characteristics from these three separate components, we combine them to form a unified and inclusive feature vector for each cluster of circRNA and drug. Finally, the relevant feature vectors and labels are inputted into a multilayer perceptron (MLP) to make predictions. The outcomes of the experiment, obtained through 5‐fold cross‐validation (5‐fold CV) and 10‐fold cross‐validation (10‐fold CV), demonstrate SNMGCDA outperforms five other state‐of‐art methods in terms of performance. Additionally, the majority of case studies have predominantly confirmed newly discovered correlations by SNMGCDA, thereby emphasizing its reliability in predicting potential relationships between circRNAs and drugs.

## 1 INTRODUCTION

Circular RNA is a novel type of RNA molecule that was first identified as a byproduct of exon scrambling during transcription. It is designed to detect mutated RNA in cancer cells that may arise from genomic translocation or rearrangement.^1^ The expression profile of circRNA differs from that of linear RNA. Generally, the expression level of circRNAs is lower, accounting for approximately 2%–10% of linear mRNA in cells. However, it is not uncommon for specific cell types to show circRNAs with expression levels that surpass those of their linear counterparts, sometimes reaching up to 10 times higher.^2^ For example, the expression level of circRNA HIP‐K3 is significantly higher compared to its linear counterpart, RNA HIP‐K3.^3^ It is noteworthy that the circRNA ciRS‐7 exhibits abundant expression in the brain, with an expression level approximately five‐fold higher than that of the housekeeping gene GAPDH.^4^ The development of high‐throughput gene sequencing technologies in recent years has made it possible to conduct in‐depth studies of the functional role of circRNA in biological systems.^5^ For instance, circRNA functions as a ‘sponge’ capable of capturing miRNA and actively modulating the developmental processes of midbrain.^6^, ^7^ Simultaneously, researchers have established the clinical significance of circRNA in the therapeutic management of diverse malignancies.^8^ The confirmation of circRNAs performing biological functions convincingly demonstrates their potential as valuable diagnostic indicators in medical environments.

Recent research findings have revealed that circRNAs exert a substantial impact on cellular drug sensitivity. For instance, in gastric cancer cells, increased levels of circAKT3 have been linked to reduced sensitivity to cisplatin in gastric cancer cells, while circ‐PVT1 has the potential to enhance resistance against paclitaxel in gastric cancer cells.^9^ Xu et al. discovered that circRNA SORE has a major part in sorafenib resistance in hepatocellular carcinoma.^10^ Additionally, Chen et al. provided evidence indicating that the expression of circRNA_0067717 is markedly rise in nasopharyngeal carcinoma cells resistant to paclitaxel treatment, suggesting a correlation between circRNA_0067717 and resistance to paclitaxel in nasopharyngeal carcinoma.^11^ These studies have provided valuable insights into the interaction between circRNA and drug action models, making important contributions to the therapeutic significance in the field of biomedical research. However, our understanding of the relationship between circRNA and drug sensitivity is still incomplete.

Due to the exorbitant costs and time‐consuming nature inherent in traditional biomedical experiments, there is an urgent imperative to develop a computationally efficient and precise methodology for foreseeing the correlations between circRNA and drug sensitivity, with the aim of mitigating both financial burdens and temporal constraints. With the continuous progress in computational power, machine learning and deep learning technologies have become widely adopted in various research fields such as analysing single‐cell multi‐omics data,^12^, ^13^, ^14^ computational toxicology,^15^, ^16^, ^17^, ^18^ metabolite–disease relationships prediction,^19^, ^20^ interaction prediction between miRNA and lncRNA or drugs,^21^, ^22^, ^23^ remote health monitoring^24^, ^25^, ^26^ and other interaction prediction problems.^27^, ^28^, ^29^, ^30^ These investigations provide strong support for computational models correlations circRNA with drug sensitivity. As an innovative study, Deng et al. first put forward a deep learning model called GATECDA^31^ for predicting the correlations between circRNA and drug sensitivity. This utilizes graph autoencoder (GATE) to derive features of circRNAs and drugs, and concatenates the extracted features linearly. Lastly, the concatenated features are passed through a deep neural network for classification, so as to obtain the associations between circRNA and drug sensitivity. Yang et al. proposed a novel neural network model called MNGACDA.^32^ In this approach, a comprehensive information matrix of circRNAs and drugs is constructed using similarity networks that incorporate various types of information. Subsequently, a node‐level attention graph autoencoder is employed to derive the representations of circRNAs and drugs from the comprehensive information matrix. Finally, the combined features are classified using the inner product decoder to determine their potential relationships. Additionally, Li et al. put forward two distinct calculation approaches, MNCLCDA and DGATCCDA, for investigating this area of study.^33^, ^34^ MNCLCDA combines similarity networks such as drug structure and circRNA sequence. It preprocesses the similarity network using random walk restart technique to reduce noise interference. Next, it utilizes a hybrid neighbourhood graph convolutional network to extract node information and enhances the model's robustness using contrastive learning. Finally, the double Laplacian‐regularized least squares approach is employed to predict the potential correlations between them. DGATCCDA utilizes a multimodal network and a graph attention network with DeepWalk perception to extract feature information and integrate this information to forecast potential relationships between circRNAs and drugs sensitivity. Moreover, Lu et al. proposed the computational framework named DHANMKF,^35^ which encodes the relationships between nodes in a bipartite multirelational heterogeneous graph based on the attention mechanism. Through the multicore fusion technology, DHNMKF combines embeddings that capture both intra‐class and inter‐class relationships, employing the double Laplacian‐regularized least squares approach to uncover the underlying connections between them. The results obtained from the above computational methods indicate the feasibility of correlation‐based approaches in predicting the associations between drug sensitivity and circRNA.

Although the above literature confirms a strong correlation between circRNA and drug sensitivity, there are still shortcomings in the application of deep learning model frameworks. The methods used for feature extraction in the models are relatively limited and lack specificity. As a result, the general architecture of the framework and the models' feature extraction techniques still have a lot of space for development. It will take more precise computational techniques to forecast the possible relationships between circRNAs and drug sensitivity.

Based on the aforementioned investigation and research, we devise an effective computational approach, named SNMGCDA, which harnesses the capabilities of deep learning, to further accurately forecast the correlations between circRNA and drug sensitivity. We combine various similarity networks of circRNAs and drugs to construct a comprehensive similarity network with information from different sources. Subsequently, three specific extraction modules are applied to obtain features. First, a sparse autoencoder module is used to extract the embedding representation of drugs. Second, a module based NMF is employed to derive features for drugs and circRNAs. In the third step, a multihead graph attention network is employed to acquire features of circRNAs. Then, element‐wise multiplication and linear concatenation are utilized to merge the feature vectors. Finally, the feature vectors and corresponding labels are fed into a classifier based on MLP for training purposes. In order to assess the performance of SNMGCDA, we utilize 5‐fold CV and 10‐fold CV. A comparison is made between our findings and those of five state‐of‐art methods. Our experimental outcomes suggest that SNMGCDA surpasses other models in multiple indicators. Furthermore, we perform pertinent case studies that demonstrate the efficacy of SNMGCDA in forecasting correlations between circRNAs and drug sensitivity.

## 2 MATERIALS AND METHODS

### 2.1 Benchmark dataset

In this study, we download circRNAs‐drug sensitivity associations data from the reference.^31^ These data are originally curated by Deng et al. and obtained from circRic database,^36^ where the drug sensitivity‐related data used is sourced from GDSC database.^37^ The dataset utilized in this investigation comprises 404 circRNAs and 250 drugs, involving a total of 80,076 associations between them. To explore the close relationships between circRNA expression and drug sensitivity, we analyse each individual circRNA using the Wilcoxon test and select relationships with a false discovery rate <0.05 as significant correlations. We extract these significant circRNA‐drug sensitivity relationships as the benchmark dataset, which include 218 drugs and 271 circRNAs, with a total of 4134 associations. We form association matrix $A\in R^{\left(271\times 218\right)}$ by utilizing these connections. For individual element within matrix *A*, $A_{\mathrm{ij}}=1$ denotes the presence of a mutual connection between circRNA *i* and drug *j*. Conversely, when $A_{\mathrm{ij}}=0$, it implies that the relationship is uncertain but could potentially exhibit correlations. Apart from collecting and organizing the relationships between circRNAs and drugs sensitivity, we also gather sequence information of circRNA host genes from NCBI Gene database,^38^ and obtain drug structure data from PubChem.^39^

### 2.2 SNMGCDA

**FIGURE 1:**
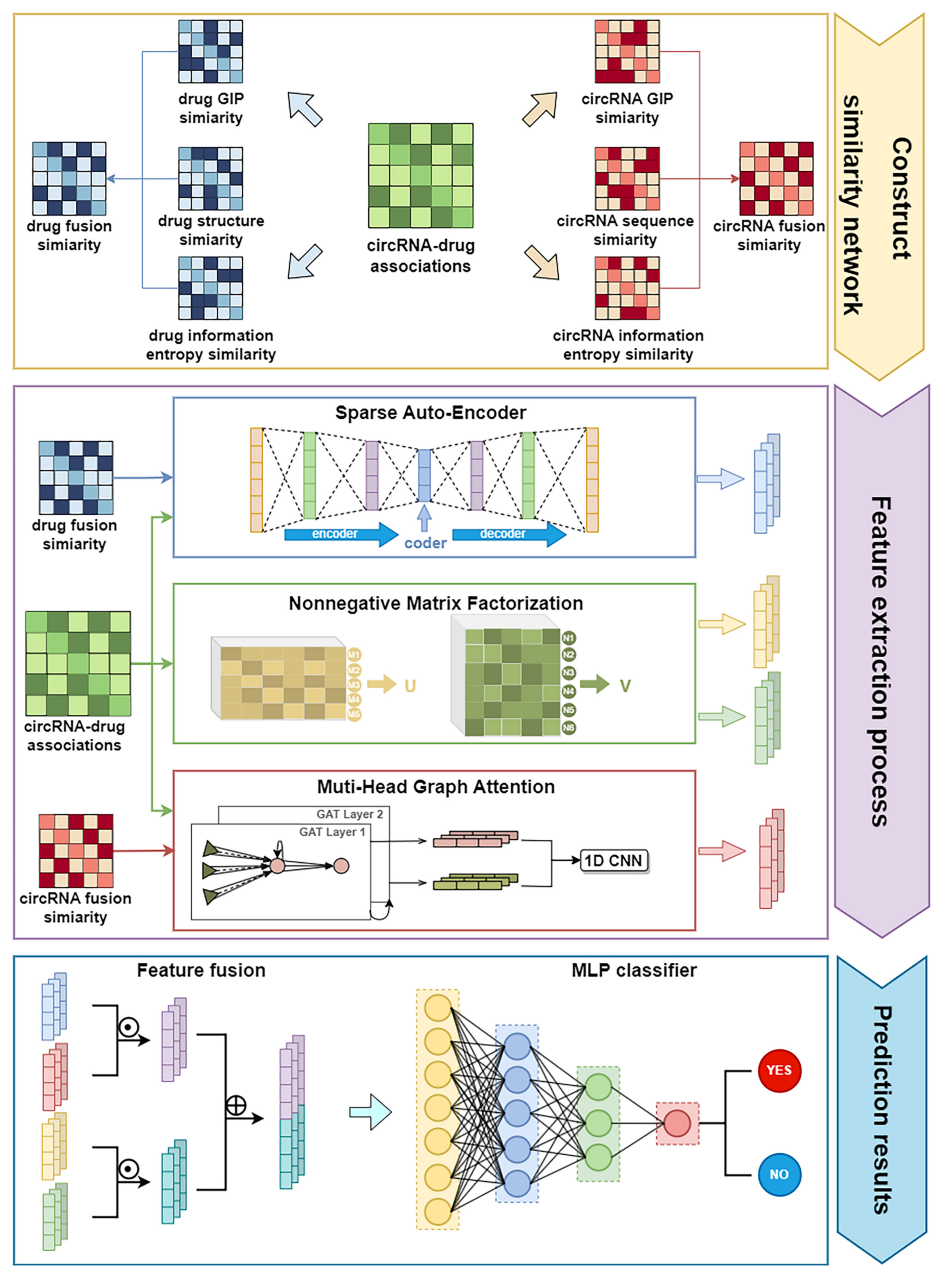
The workflow of SNMGCDA.

The framework of SNMGCDA is illustrated in Figure 1. Initially, we add biological information by computing structural similarity, Gaussian kernel similarity and information entropy similarity networks for drugs, and sequence similarity, Gaussian kernel similarity and information entropy similarity networks for circRNAs. Subsequently, three distinct modules are employed for feature extraction: the sparse autoencoder is utilized to acquire drug features, the NMF module simultaneously obtains features of circRNAs and drugs while leveraging the multi‐head graph attention network specifically for acquiring circRNA features. Next, through element‐by‐element multiplication and linear splicing, we fuse the feature vectors together with their corresponding labels as inputs into MLP classifier for training purposes. Consequently, within MLP, we obtain predictive results pertaining to sensitivity correlations between circRNAs and drugs.

### 2.3 Similar network construction

#### 2.3.1 Structural similarity of drugs

The inherent structural characteristics of drugs determine their functional role. Hence, the similarity between them can be evaluated by analysing the structure of drugs. After obtaining the structural information from PubChem database, our initial step involves utilizing RDKit^40^ to generate topological fingerprints for each drug. Subsequently, we compute the Tanimoto coefficient (a representation that encodes molecular structure into a bit vector) to measure the structural similarity between drugs:

(1) $$L\left(d_{A},d_{B}\right)=\frac{d_{A}\cdot d_{B}}{{\left|d_{A}\right|}^{2}+{\left|d_{B}\right|}^{2}-d_{A}\cdot d_{B}}$$

Here, $d_{A}$ and $d_{B}$ represent the topological fingerprint vectors of drugs *A* and *B*, respectively. $d_{A}\cdot d_{B}$ is the dot product of these two vectors, ${\left|d_{A}\right|}^{2}$ and ${\left|d_{B}\right|}^{2}$ are the number of 1 in each vector, respectively. Finally, we establish a matrix $\mathrm{DSS}\in R^{\left(M\times M\right)}$ (where *M* is the total number of drugs) that represents the structural similarity of drugs, and *DSS* contains the similarity score between each drug and all other drugs.

#### 2.3.2 Sequence similarity of circRNAs host genes

To assess the similarity between circRNAs, we employ a methodology akin to that proposed by Deng et al.^31^ Specifically, we regard the similarity of host genes among circRNAs as an indicator of their own similarity. With such a methodology, we can quantify the similarity among circRNAs by calculating the Levenshtein distance of their host gene sequences. To accomplish this, we employ the ratio function provided by the Levenstein‐package in Python for distance calculation. The formula is presented as follows:

(2) $$\begin{array}{c} {\mathrm{lev}}_{a,b}\left(i,j\right)=\left\{\begin{array}{c} \max\left(i,j\right)\;\text{if}\;\min\left(i,j\right)=0\\ \min\left\{\begin{array}{c} {\mathrm{lev}}_{a,b}\left(i-1,j\right)+1\\ {\mathrm{lev}}_{a,b}\left(i,j-1\right)+1\\ {\mathrm{lev}}_{a,b}\left(i-1,j-1\right)+1_{\left(a_{i}\neq b_{j}\right)} \end{array}\right.\text{otherwise} \end{array}\right. \end{array}$$

Here, *a* and *b* represent the circRNA sequences to be compared, *i* and *j* denote the current positions of two sequences *a* and *b*, respectively. Finally, we establish a circRNA sequence similarity matrix $\mathrm{CSS}\in R^{\left(N\times N\right)}$ (where *N* represents the total number of circRNAs), which encompasses the similarity scores between each circRNA and all other circRNAs.

#### 2.3.3 Gaussian nuclear similarity for drugs or circRNAs

The Gaussian kernel similarity of drugs is determined by applying the Gaussian kernel function to compute the similarity score between two drugs. In the drug network, drugs exhibiting similar properties often exhibit close relationships with one another. To calculate Gaussian interaction spectrum kernel similarity (*DGIP*) between drug $d_{i}$ and drug $d_{j}$, we employ the subsequent Gaussian kernel formula:

(3) $$\text{DGIP}\left(d_{i},d_{j}\right)=\exp\left(-\omega_{d}∥\mathrm{IP}\left(d_{i}\right)-\mathrm{IP}\left(d_{j}\right){∥}^{2}\right)$$

(4) $$\omega_{d}=\frac{\omega_{d}^{\prime }*M}{\sum_{i=1}^{n^{d}}\mathrm{IP}{\left(d_{i}\right)}^{2}}$$

Here *i* represents the number of columns corresponding to drugs in the matrix *A*, $\mathrm{IP}\left(d_{i}\right)$ and $\mathrm{IP}\left(d_{j}\right)$ represent the corresponding columns and rows of drug $d_{i}$ and drug $d_{j}$ in the matrix *A*, respectively.

The normalized kernel bandwidth $\omega_{d}$ is a crucial parameter that determines sample density in the space of characteristics and manages the breadth of the similarity measure. Choosing the right bandwidth value can achieve a harmonious blend of smoothness and sharpness, resulting in enhanced classification outcomes. We can adjust $\omega_{d}$ by updating the normalized bandwidth $\omega_{d}^{\prime }$, typically setting $\omega_{d}^{\prime }$ to 1.

In a similar vein, we calculate the Gaussian interaction spectrum kernel similarity (*CGIP*) for circRNA sequence $c_{i}$ and circRNA sequence $c_{j}$ by the above method:

(5) $$\text{CGIP}\left(c_{i},c_{j}\right)=\exp\left(-\omega_{c}∥\mathrm{IP}\left(c_{i}\right)-\mathrm{IP}\left(c_{i}\right){∥}^{2}\right)$$

(6) $$\omega_{c}=\frac{{\omega_{c}^{\prime }}^{*}N}{\sum_{i=1}^{n^{c}}\mathrm{IP}{\left(c_{i}\right)}^{2}}$$

Where $\mathrm{IP}\left(c_{i}\right)$ and $\mathrm{IP}\left(c_{j}\right)$ represent the number of columns and rows corresponding to circRNA $c_{i}$ and circRNA $c_{j}$, respectively. The normalized kernel bandwidth, denoted as $\omega_{c}$, can be adjusted using the updated normalized bandwidth $\omega_{c}^{\prime }$. The value of $\omega_{c}^{\prime }$ is usually set to 1.

#### 2.3.4 Similarity of circRNAs or drugs based on information entropy

The initial introduction of the notion of information entropy can be attributed to Claude Shannon, who proposed its definition as a measure of the amount of information and used it to describe uncertainty in information transmission. Today, with the advancement of computer science, information entropy has become a fundamental concept in data compression, encryption and network communication. For instance, Huffman coding algorithms utilize the principle of information entropy. In a prior investigation, Li et al. employed information entropy as a metric to quantify the similarity between miRNA and disease.^41^ In this study, we integrate the shared information of circRNAs and drugs, and assess the similarity between circRNA sequences and drugs through computation of information entropy. As an example, let's consider the computation of circRNA sequence similarity using information entropy: $I_{c}^{A}$ symbolizes the drug set related to circRNA *A*, $I_{c}^{A}=\left\{I_{c}^{A}\left(1\right),I_{c}^{A}\left(2\right),\ldots ,I_{c}^{A}\left(n_{a}\right)\right\}$. Similarly, $I_{c}^{B}$ symbolizes the drug set $I_{c}^{B}=\left\{I_{c}^{B}\left(1\right),I_{c}^{B}\left(2\right),\ldots ,I_{c}^{B}\left(n_{b}\right)\right\}$ related to circRNA *B*. Among them, $n_{a}$ and $n_{b}$ correspond to the number of drug types associated with circRNA *A* and circRNA *B*, respectively. The calculation method for determining the similarity in information entropy between circRNA sequences and drugs can be expressed as follows:

(7) $$F\left(I_{c}^{A}\right)=-\sum_{i=1}^{n_{\mathrm{na}}}p\left(I_{c}^{A}\left(i\right)\right)\log_{2}\left(p\left(I_{c}^{A}\left(i\right)\right)\right)$$

(8) $$p\left(I_{c}^{A}\left(i\right)\right)=\frac{n\left(I_{c}^{A}\left(i\right)\right)}{\text{AN}}$$

*AN* is the number of correlations between all circRNAs and drugs, and $n\left(I_{c}^{A}\left(i\right)\right)$ is the number of known associations of all circRNAs in the drug set where the *i‐*th drug is known to be associated with circRNA *A*. The information entropy based similarity (*CSIE*) between circRNA *A* and circRNA *B* is calculated as follows:

(9) $$\text{CSIE}\left(A,B\right)=\frac{2\times F\left(I_{c}^{A}\cap I_{c}^{B}\right)}{F\left(I_{c}^{A}\right)+F\left(I_{c}^{B}\right)}$$

$I_{c}^{A}\cap I_{c}^{B}$ denotes the intersection of related drugs shared by circRNA *A* and circRNA *B*. Similarly, we can derive the drugs similarity based on information entropy (*DSIE*) by computing the information entropy of drug and circRNA sequences as well as their mutual information.

### 2.4 Integrated circRNAs or drugs similarity networks

In contrast to the conventional linear ensemble approach,^22^ we adopt a non‐linear strategy to integrate circRNAs or drugs similarity networks from diverse sources, with the goal of effectively capturing both overlapping and complementary insights across multiple data sets,^42^ thereby reducing noise. These noises could potentially arise from inherent inaccuracies in data measurement and variations in sample collection, or they might be attributed to the introduction of deviation noises during the feature extraction procedure. Through integration, we obtain a fused circRNAs or drugs similarity network. Within this specific segment, we introduce the process of circRNAs similarity network integration in details.

Firstly, it is essential to standardize each circRNA network. Taking *CSS* as an exemplar, the normalization procedure is outlined as follows:

(10) $${\mathrm{SM}}_{\mathrm{CSS}}\left(i,j\right)=\left\{\begin{array}{lll} \frac{\mathrm{CSS}\left(i,j\right)}{2^{*}\sum_{k\neq i}\mathrm{CSS}\left(i,k\right)}, & j\neq i & \\ \;\frac{1}{2}, & j=i & \end{array}\right.$$

After normalizing CSS, ${\mathrm{SM}}_{\mathrm{CSS}}$ is obtained. All the elements on the diagonal are assigned a value of 1/2, while ensuring that the sum of each row's elements is equal to 1. Similarly, we can obtain ${\mathrm{SM}}_{\text{CGIP}}$ and ${\mathrm{SM}}_{\text{CSIE}}$. Subsequently, employing the K nearest neighbour algorithm enables us to calculate the local affinity ${\mathrm{SKN}}_{\mathrm{CSS}}$ between circRNA *i* and circRNA *j*:

(11) $${\mathrm{SKN}}_{\mathrm{CSS}}\left(i,j\right)=\left\{\begin{array}{l} \frac{\mathrm{CSS}\left(i,j\right)}{\sum_{k\in N_{i}}\mathrm{CSS}\left(i,k\right)},\;j\in N_{i}\\ \;0,\;\text{otherwise}\; \end{array}\right.$$

Here $N_{i}$ represents the K nearest neighbours of a given node, where it is calculated as one‐tenth of the total number of circRNAs. The basic principle of this method is that when the distance between the two is closer, the similarity is higher. To guarantee precision, we assign a similarity score of 0 to distant nodes that are distant from the given node. Similarly, employing the aforementioned method allows us to obtain ${\mathrm{SKN}}_{\text{CGIP}}$ and ${\mathrm{SKN}}_{\text{CSIM}}$.

Subsequently, each similarity network is updated by an iterative process:

(12) $${\mathrm{SM}}_{\mathrm{CSS}}^{\left(t\right)}={\mathrm{SKN}}_{\mathrm{CSS}}\times \left(\frac{\sum_{k\neq \mathrm{CSS}}SM_{k}^{\left(t-1\right)}}{d-1}\right)\times {\left({\mathrm{SKN}}_{\mathrm{CSS}}\right)}^{T}$$

(13) $${\mathrm{SM}}_{\text{CGIP}}^{\left(t\right)}={\mathrm{SKN}}_{\text{CGIP}}\times \left(\frac{\sum_{k\neq \text{CGIP}}SM_{k}^{\left(t-1\right)}}{d-1}\right)\times {\left({\mathrm{SKN}}_{\text{CGIP}}\right)}^{T}$$

(14) $${\mathrm{SM}}_{\text{CSIM}}^{\left(t\right)}={\mathrm{SKN}}_{\text{CSIM}}\times \left(\frac{\sum_{k\neq \text{CSIM}}SM_{k}^{\left(t-1\right)}}{d-1}\right)\times {\left({\mathrm{SKN}}_{\text{CSIM}}\right)}^{T}$$

In this context, *d* symbolizes the number of distinct circRNAs similarity networks. Specifically, there are three such networks denoted by *d* = 3. The variable *k* signifies the currently selected circRNAs similarity network, with possible values being CSS, CGIP or CSIE. *T* denotes the total number of iterations performed. ${\mathrm{SM}}_{\mathrm{CSS}}^{\left(t\right)}$, ${\mathrm{SM}}_{\text{CGIP}}^{\left(t\right)}$ and ${\mathrm{SM}}_{\text{CSIM}}^{\left(t\right)}$ represent the state matrices of ${\mathrm{SM}}_{\mathrm{CSS}}$, ${\mathrm{SM}}_{\text{CGIP}}$ and ${\mathrm{SM}}_{\text{CSIM}}$ after *t* iterations, respectively. Following each iteration, it is necessary to renormalize and calculate the comprehensive similarity network anew. The comprehensive similarity network *SM* can be expressed as follows:

(15) $$\mathrm{SM}=\frac{{\mathrm{SM}}_{\mathrm{CSS}}^{\left(t\right)}+{\mathrm{SM}}_{\text{CGIP}}^{\left(t\right)}+{\mathrm{SM}}_{\text{CSIE}}^{\left(t\right)}}{3}$$

The completion of the normalization calculation iteration for the comprehensive similarity network is determined by meeting condition $\frac{∥{\mathrm{SM}}_{k}^{\left(t\right)}-{\mathrm{SM}}_{k}^{\left(t-1\right)}∥}{∥{\mathrm{SM}}_{k}^{\left(t-1\right)}∥}<10^{-6}$. Subsequently, matrix transformation by $SM^{\prime }=\frac{\mathrm{SM}+{\mathrm{SM}}^{T}}{2}$ converts *SM* into a symmetric matrix denoted as $SM^{\prime }$. This final symmetric matrix represents our integrated matrix. Similarly, we can apply the aforementioned steps to obtain the comprehensive similarity network $SD^{\prime }$ for drugs.

### 2.5 Feature extraction

#### 2.5.1 Drugs feature extraction based on sparse autoencoder

The sparse autoencoder is an efficient unsupervised machine learning algorithm that focuses on extracting valuable features from data. By cleverly integrating the structural and sparsity principles of autoencoders, it can achieve data compression and feature extraction while preserving important information.^43^ The sparse autoencoder continuously optimizes network parameters by minimizing the error between input and output, resulting in an effective model for accurately representing the original data. This model comprises two main components: an encoder and a decoder, which introduce the crucial concept of sparsity. Specifically, a sparse penalty term is incorporated into the hidden layer to enforce activation of only a few neurons for representing input data. Such a sparse activation pattern facilitates extraction of more significant and meaningful features.

The module of the sparse autoencoder is a sequential model that consists of two encoders and decoders, which are constructed using several linear layers and non‐linear activation functions. The encoder consists of four layers, with neuron counts of 400, 350, 256 and 128 in each layer, respectively. Similarly, the decoder is comprised of four layers with neuron counts arranged as follows: 128, 256, 350 and finally 400. We utilize the output from the encoder layer as the drug's feature representation. The loss function in this module encompasses two components: reconstruction loss and sparsity loss. The reconstruction loss is computed by comparing the discrepancy between the output of the autoencoder (i.e. reconstructed input) and the original input. Binary cross entropy loss is employed in this module:

(16) $$L_{\text{reconstruction}}=-\frac{1}{M}\sum_{i=1}^{M}\left[y_{i}\cdot \log\left({\hat{y}}_{i}\right)+\left(1-y_{i}\right)\cdot \log\left(1-{\hat{y}}_{i}\right)\right]$$

Where $y_{i}$ is the true value of the *i*‐th drug, and ${\hat{y}}_{i}$ is the predicted value of the *i*‐th drug sample reconstruction by the autoencoder.

The sparsity loss is employed for acquiring a sparse feature representation in this model. Specifically, the sparsity loss is computed by evaluating the mean squared error of the layer weights within a specific layer. The formula in question can be represented as follows:

(17) $$L_{\text{sparse}}=\alpha \cdot \mathrm{MSE}\left(x,\beta \right)=\alpha \cdot \frac{1}{K}\sum_{j=1}^{K}{\left(x_{j}-\beta \right)}^{2}$$

Here, α represents the weight for sparsity regularization, *K* denotes the overall count of elements within the weight tensor and $x_{j}$ indicates the activation value of the *j*‐th element in the coding layer's weight tensor. β signifies the sparsity target, which reflects the desired level of sparse activation. Ultimately, we can express the overall loss as: $L_{\text{total}}=L_{\text{reconstruction}}+L_{\text{sparse}}$. The loss function plays a crucial role in updating model weights during training by optimizing it to enable effective input reconstruction while preserving sparsity.

#### 2.5.2 CircRNAs feature extraction based on multihead graph attention

We utilize a multihead graph attention module to acquire the feature representation of circRNAs. Within this module, we use a two‐layer graph neural network consisting of three attention heads, each containing 64 neurons. This module enables us to capture the characteristics of individual circRNA nodes while preserving their embedded graph structure information. Regarding the initial input feature matrix for this module, our construction method is as follows:

(18) $$X=\left[\begin{array}{cc} {\mathrm{SM}}^{\prime } & A\\ A^{T} & 0 \end{array}\right]$$

The feature matrix *X* undergoes a linear transformation through a trainable weight matrix *W*, resulting in the mapping of node features to a high‐dimensional space. This transformation can be mathematically expressed for each header as follows:

(19) H=XW

Here, $X\in {\mathbb{R}}^{N\times F}$ is the input characteristic matrix, *N* is the number of nodes and *F* is the characteristic number of each node. $W\in {\mathbb{R}}^{F\times F^{\prime }}$ is the weight matrix of the linear transformation ($F^{\prime }$ is the dimension of the output feature, usually $F^{\prime }=\frac{F}{N_{\text{heads}}}$), and $H\in {\mathbb{R}}^{N\times F^{\prime }}$ is the transformed feature matrix.

The attention coefficient is then computed by utilizing a trainable vector a and applying a nonlinear function (LeakyReLU). The computation of the attention coefficient for each head can be formulated as follows:

(20) $$e_{\mathrm{ij}}=\text{LeakyReLU}\left(a^{T}\left[h_{i}∥h_{j}\right]\right)$$

The attention coefficient of node *j* to node *i*, denoted as $e_{\mathrm{ij}}$, is calculated based on the feature vectors $h_{i}$ and $h_{j}$. The symbol ∥ represents vector concatenation, while the parameter vector $a\in {\mathbb{R}}^{2F^{\prime }}$ is utilized for computing the attention coefficient.

Secondly, we employ a normalization technique to ensure that the attention coefficients of all input neighbour nodes for each node *i* sum up to 1, thereby enhancing the interpretability and stability of SNMGCDA:

(21) $$\alpha_{\mathrm{ij}}=\text{softmaxj}\left(e_{\mathrm{ij}}\right)=\frac{\exp\left(e_{\mathrm{ij}}\right)}{\sum_{k\in N\left(i\right)}\exp\left(e_{\mathrm{ik}}\right)}$$

Where $N\left(i\right)$ is the set of neighbour nodes of node *i*. Finally, we use the activation function σ to aggregate each node according to the attention coefficient and the characteristics of the neighbour nodes to acquire the final node representation:

(22) $$Hi^{\prime }=\sigma \left(\sum_{j\in N\left(i\right)}\alpha_{\mathrm{ij}}h_{j}\right)$$

#### 2.5.3 Drugs and circRNAs feature extraction based on NMF

In 1999, Lee and Seung et al.^44^ put forward a new matrix factorization (NMF) method. Subsequently, Ding et al. used NMF to get significant features between miRNAs and diseases.^42^ The NMF technique is a matrix decomposition method that aims to decompose a non‐negative matrix $C_{m\times n}$ into two non‐negative matrices $U_{m\times k}$ and $V_{k\times n}$. By minimizing the difference between the product of $U_{m\times k}$ and $V_{k\times n}$, an effective decomposition of $C_{m\times n}$ can be achieved. In this context, we refer to $U_{m\times k}$ as the base matrix and $V_{k\times n}$ as the coefficient matrix.

After performing factorization, the column vector of matrix *C* can be expressed as a linear combination of the entire set of column vectors in matrix *U*, where the weight of each column vector is determined by the elements of the corresponding column vector of matrix *V*. When *k* is less than *M* and satisfies the condition $\left(M+N\right)\times k<\mathrm{MN}$, it becomes easier to capture shared structures within data and obtain a lower‐dimensional representation. To ensure smoothness in matrices $U_{M\times k}$ and $V_{k\times N}$, we utilize Tikhonov regularization L_2_ method for processing:

(23) $$\min_{U\geq 0,V\geq 0}∥W⊙\left(C-\mathrm{UV}\right){∥}_{F}^{2}+\lambda_{1}∥U{∥}_{F}^{2}+\lambda_{2}∥V{∥}_{F}^{2}$$

The matrix *W* is assigned the same values as *A*, where ⊙ denotes element‐wise multiplication. $\lambda_{1}$ and $\lambda_{2}$ are regularization coefficients with a fixed value of 0.01, while *k* is set to 128. Here ${\left\|\cdot \right\|}_{F}$ represents the Frobenius norm, which quantifies the sum of squared differences between elements in a matrix.

Then we use the iterative solution method in NMF to update the matrices *U* and *V*:

(24) $$U_{i,k}^{\left(n+1\right)}\leftarrow U_{i,k}^{n}\frac{{\left(\left(W⊙C\right)V^{T}\right)}_{i,k}}{{\left(\left(W⊙\left(\mathrm{UV}\right)\right)V^{T}+\left(\frac{\lambda_{1}}{2}\right)J\right)}_{i,k}}$$

(25) $$V_{k,j}^{\left(n+1\right)}\leftarrow V_{k,j}^{n}\frac{{\left(U^{T}\left(W⊙C\right)\right)}_{k,j}}{{\left(U^{T}\left(W⊙\left(\mathrm{UV}\right)\right)+\left(\frac{\lambda_{2}}{2}\right)J\right)}_{k,j}}$$

The NMF module is configured with 500 iterations for updating the *U* and *V* matrices, resulting in the acquisition of a circRNA characteristic matrix $U_{271\times 128}$ and a drug characteristic matrix $V_{218\times 128}^{T}$.

### 2.6 Feature fusion

In summary, SNMGCDA employs three modules to extract a total of 256 features representing circRNAs and drugs. Initially, the sparse autoencoder module extracts 128 drug features, while the multihead map attention module obtains 128 circRNAs features. Furthermore, the NMF module yields an additional set of 128 drug features and 128 circRNAs features. Additionally, we combine the feature representations from the sparse autoencoder and multihead graph attention modules using element‐by‐element multiplication to obtain a fused representation that captures both drug and circRNA characteristics. Subsequently, we linearly concatenate this fused representation with the feature representation from the NMF module to create a comprehensive long feature representation resulting in each circRNA‐drug association being represented by a 256‐dimensional feature vector. If there is a known relationship between a specific circRNA and drug in our dataset, we assign its corresponding label as 1, otherwise it is assigned as 0. Finally, these feature vectors along with their respective labels are utilized for training an MLP classifier to obtain accurate classification results. SNMGCDA integrates multiple modules effectively enabling high‐dimensional representation of circRNA‐drug correlations leading to improved classification accuracy.

### 2.7 MLP

MLP is a feed‐forward artificial neural network model composed of multilayer nodes and neurons.^45^ After receiving the input, each neuron uses a non‐linear activation function to convert these signals and then transmits them to the subsequent layer. Upon receiving the input, each neuron transforms these signals using a non‐linear activation function before transmitting them to the subsequent layer. MLP is a widely used supervised learning algorithm for classification tasks. In SNMHCAD, we use a four‐layer fully connected MLP, each of which consists of different numbers of neurons: 256, 128, 64 and 1.

To rectify the disparity between positive and negative samples in the benchmark dataset, we adopt a balanced 1:1 sampling approach. This involves selecting all positive samples along with an equal number of randomly selected negative samples. Following this, we proceed to train a MLP model using the features and corresponding labels from the training set. The objective is to leverage the MLP's outcomes as final predictions for the test set.

## 3 RESULTS

### 3.1 Performance evaluation

We evaluate the performance of SNMGCDA by using 5‐fold CV and 10‐fold CV techniques. Using the same evaluation steps throughout the experiment, a comprehensive evaluation of SNMGCDA is obtained. In order to evaluate the effectiveness of SNMGCDA, several commonly used binary indicators are used. The precise definitions of these indicators are given below:

(26) $$\mathrm{TPR}=\frac{\mathrm{TP}}{\mathrm{TP}+\mathrm{FN}}$$

(27) $$\mathrm{FPR}=\frac{\mathrm{FP}}{\mathrm{TN}+\mathrm{FP}}$$

(28) $$\text{Precision}=\frac{\mathrm{TP}}{\mathrm{TP}+\mathrm{FP}}$$

(29) $$\text{Recall}=\frac{\mathrm{TP}}{\mathrm{TP}+\mathrm{FN}}$$

(30) $$\text{Specificity}=\frac{\mathrm{TN}}{\mathrm{TN}+\mathrm{FP}}$$

(31) $$\text{Accuracy}=\frac{\mathrm{TP}+\mathrm{TN}}{\mathrm{TP}+\mathrm{TN}+\mathrm{FP}+\mathrm{FN}}$$

(32) $$F1=2\times \frac{\text{Precision}\times \mathrm{TPR}}{\text{Precision}+\mathrm{TPR}}$$

In the context of problems involving the classification of binary, the model predicts samples and categorizes them as either positive or negative instances. *TP* and *TN* denote samples that are correctly identified as positive and negative, while *FP* and *FN* refer to samples that are incorrectly classified as positive and negative. Precision refers to the probability that all positively classified instances by the model are indeed positive cases, whereas Recall pertains to the probability that actual positive cases are correctly identified as such.

ROC curve, also known as the sensitivity curve, is a visual representation of binary classification performance. It illustrates the relationship between false positive rate (FPR) and true positive rate (TPR). FPR represents the proportion of negative samples incorrectly classified as positive cases, while TPR indicates the proportion of correctly predicted positive samples. Evaluating classification effectiveness can be done by calculating the area under this curve (AUC), where larger AUC values indicate better performance. Another metric used for assessing model performance in imbalanced datasets is AUPR, which measures the area under the precision‐recall curve. In this plot, Precision is shown on the horizontal axis and Recall on the vertical axis. Higher AUPR values correspond to superior model performance. We meticulously select and configure the experimental parameters as presented in Table 1. When integrating the circRNA similarity network, we set $k_{1}$ to 27, which corresponds to one‐tenth of the total number of circRNAs. Similarly, for integrating the drug similarity network, we designate $k_{2}$ as 21, representing one‐tenth of all drugs. The Adam optimizer is chosen for the sparse autoencoder module. In the multihead graph attention module, a maximum learning rate of 0.001 is employed. Regarding the NMF module, $\lambda_{1}=\lambda_{2}=0.01$ and k=128 are adopted as regularization coefficients. Further details regarding the hyperparameters of SNMGCDA can be found in Table 1.

**TABLE 1:** The main parameters of SNMGCDA.

Structure	Parameters	Value
KNN for circRNA	k_1_	27
KNN for drug	k_2_	21
Sparse autoencoder	Encoder/decoder layer number	4
	Sparsity weight	e^−3^
	Neuron numbers of encoder layer	400, 350, 256, 128
	Neuron numbers of decoder layer	128, 256, 350, 400
	Optimizer	Adam
	Loss	mse + sparse loss
	Epochs	50
	Batch_size	128
Mutihead GAE	Hidden layer number	2
	Number of heads per layer	3
	Weight decay	5e^−3^
	Feature embedding size per layer	64
	Learning rate	max:e^−3^
NMF	$\lambda_{1}$	e^−2^
	$\lambda_{2}$	e^−2^
	k	128

### 3.2 Comparison with previous methods

In this section, we compare SNMGCDA with other computational methods in the relevant field to comprehensively evaluate the performance of our model. Specifically, we benchmark our model against five well‐established approaches, namely GATECDA,^31^ MNGACDA,^32^ MNCLCDA,^33^ DGATCCDA^34^ and ADPMDA.^46^ It is worth noting that while in addition to ADPMDA methods have been employed for predicting miRNA‐disease correlations, the remaining four methods have been utilized for foreseeing circRNA‐drug sensitivity associations.

- GATECDA: Graph autoencoders are employed to extract features from circRNA and drug networks, respectively. Subsequently, a fully connected layer is utilized to identify relationships between circRNAs and drugs sensitivity.
- MNGACDA: A multimodal network is constructed using a combination of graph autoencoder and attention mechanism to investigate potential relationships between circRNAs and drugs sensitivity.
- MNCLCDA: The random walk restart technique is employed to construct a similar network, while the mixed neighbourhood graph convolution network is utilized for node information acquisition in order to discern correlations between ring structures and drug sensitivity.
- DGATCCDA: A graph attention network, which is enhanced with DeepWalk knowledge, is utilized to capture feature details and combine them for the foreseeing of potential connections between circRNAs and drugs.
- ADPMDA: The utilization of adaptive deep propagation graph neural network enables the acquisition of embedded nodes, dynamic adjustment of local and global node information, and prediction of miRNA‐disease connections using a multilayer perceptron.

**FIGURE 2:**
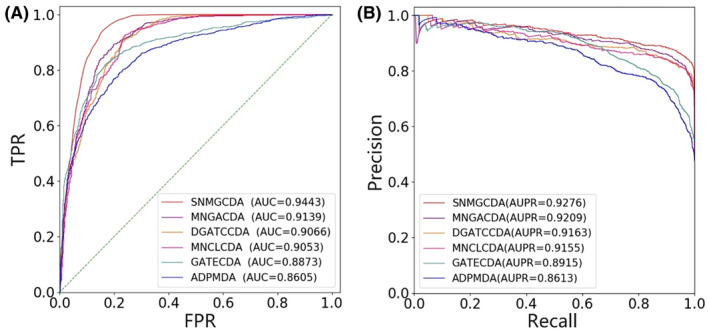
ROC and PR curves of SNMGCDA and other methods under 5‐fold CV on benchmark dataset.

**FIGURE 3:**
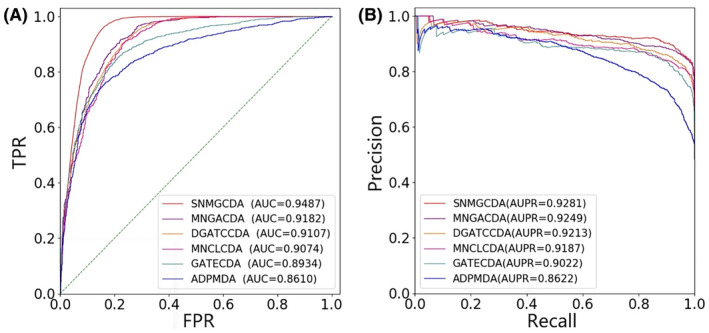
ROC and PR curves of SNMGCDA and other methods under 10‐fold CV on benchmark dataset.

We conduct 5‐fold CV and 10‐fold CV experiments on the dataset to assess the predictive performance. All approaches are evaluated under identical experimental conditions, employing the suggested optimal parameters recommended in the study. In 5‐fold CV experiment, as depicted in Figure 2A, SNMGCDA exhibits an average AUC value of 0.9443. Notably, it outperforms other methods by a margin of 3.04% (MNGACDA), 3.77% (DGATCCDA), 3.90% (MNCLCDA), 5.70% (GATECDA) and 8.38% (ADPMDA). The corresponding AUPR results are presented in Figure 2B, where SNMGCDA achieves an average AUPR value of 0.9276, surpassing other methods by increments of 0.67% (MNGACDA), 1.13% (DGATCCDA), 1.21% (MNCLCDA), and notably higher margins of improvement for GATECDA at 3.16%, and ADPMDA at 6.63%. Furthermore, the values of other performance indicators, such as F1‐Score value, Accuracy, Precision, Recall and Specificity are presented in Table 2. SNMGCDA achieves the highest results among the five methods with values of 0.8968, 0.8913, 0.9435, 0.8389 and 0.8552, respectively. In 10‐fold CV experiment, as shown in Figure 3A, the average AUC value of SNMGCDA is 0.9487, which is 3.05% (MNGACDA), 3.80% (DGATCCDA), 4.13% (MNCLCDA), 5.53% (GATECDA) and 8.77% (ADPMDA) higher than other methods, respectively. The results of the AUPR can be observed in Figure 3B. It is evident that AUPR score achieved by SNMGCDA stands at an impressive 0.9281, which is 0.32% (MNGACDA), 0.68% (DGATCCDA), 0.94% (MNCLCDA), 2.59% (GATECDA) and 6.59% (ADPMDA) higher than other methods, respectively. The values of other performance indicators are shown in Table 3. The best results obtained by SNMGCDA are 0.9052, 0.8988, 0.9412, 0.8416 and 0.8596, respectively, which are higher than the other five methods. These extensive findings demonstrate that SNMGCDA outperforms the five state‐of‐art approaches.

**TABLE 2:** Comparative analysis of SNMGCDA and other methods under 5‐fold CV on benchmark dataset.

Method	AUC	AUPR	F1	ACC	Recall	Spe	Pre
SNMGCDA	0.9443	0.9276	0.8968	0.8913	0.9435	0.8389	0.8552
MNGACDA	0.9139	0.9209	0.8472	0.8424	0.8723	0.8155	0.8247
DGATCCDA	0.9066	0.9163	0.8426	0.8408	0.8586	0.8322	0.8369
MNCLCDA	0.9053	0.9155	0.8394	0.8367	0.8547	0.8303	0.8356
GATECDA	0.8873	0.8915	0.8224	0.8186	0.8404	0.7966	0.8054
ADPMDA	0.8605	0.8613	0.8112	0.8023	0.8182	0.7945	0.8022

**TABLE 3:** Comparative analysis of SNMGCDA and other methods under 10‐fold CV on benchmark dataset.

Method	AUC	AUPR	F1	ACC	Recall	Spe	Pre
SNMGCDA	0.9487	0.9281	0.9052	0.8988	0.9412	0.8416	0.8596
MNGACDA	0.9182	0.9249	0.8519	0.8498	0.8646	0.8350	0.8401
DGATCCDA	0.9107	0.9213	0.8436	0.8453	0.8611	0.8407	0.8421
MNCLCDA	0.9074	0.9187	0.8424	0.8415	0.8603	0.8389	0.8418
GATECDA	0.8934	0.9022	0.8286	0.8273	0.8367	0.8172	0.8227
ADPMDA	0.8610	0.8622	0.8093	0.8120	0.8326	0.7876	0.7954

### 3.3 Comparison with other classifiers

**FIGURE 4:**
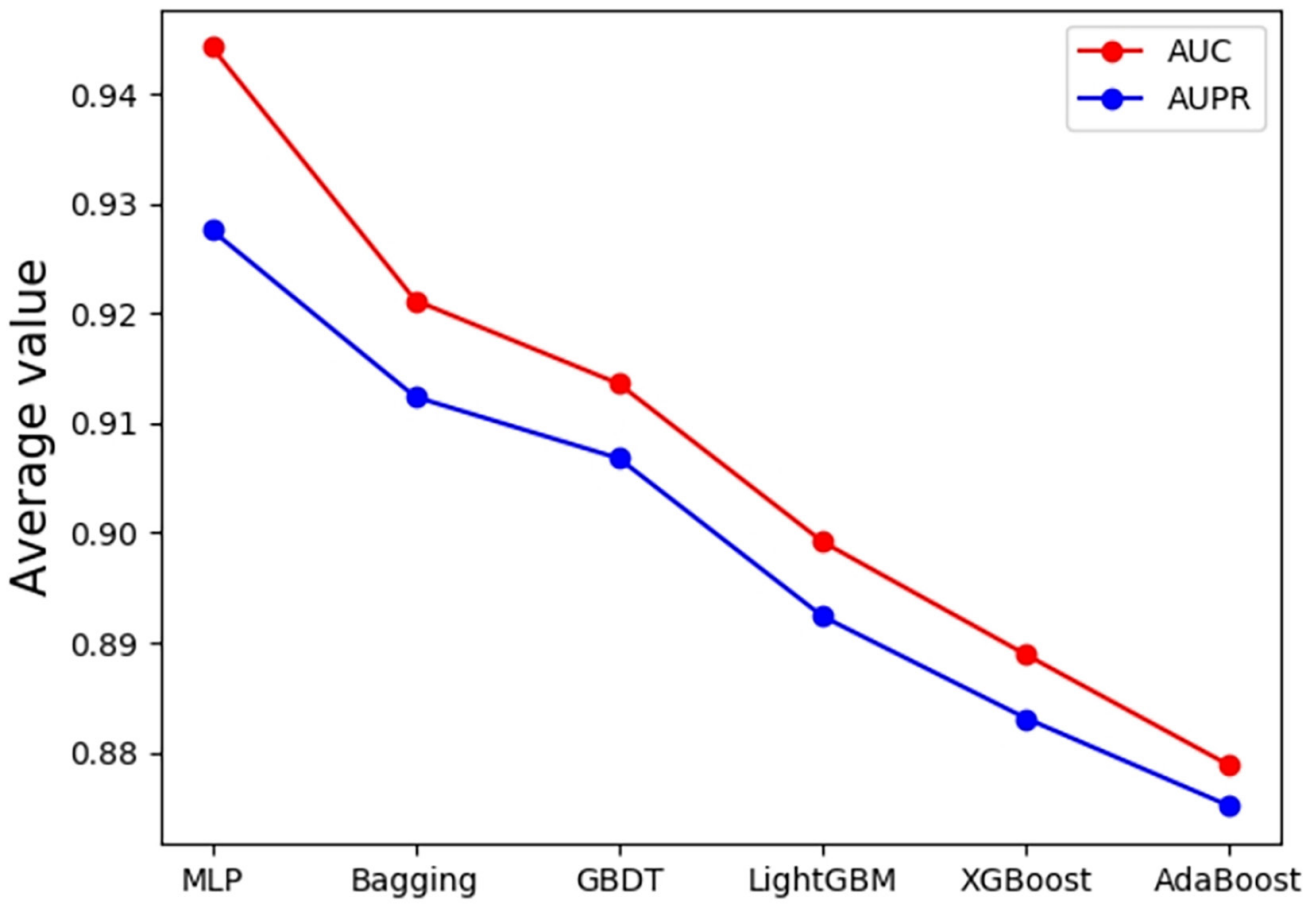
AUC and AUPR values of MLP compared with other popular classifiers on the benchmark dataset.

To assess the efficacy of MLP, we perform a comparative analysis through 5‐fold CV experiments with other commonly used classifiers, namely Bagging, AdaBoost, GBDT, XGBoost and LightGBM. The findings are displayed in Figure 4. Specially, MLP achieves superior performance compared to other classifiers in terms of AUC and AUPR scores, with values reaching 0.9443 and 0.9246, respectively, indicating its superior ability to distinguish positive and negative samples more accurately while maintaining higher precision for positive samples. The second‐best performer is Bagging classifier scoring AUC of 0.9212 and AUPR score of 0.9124 whereas AdaBoost performs poorly with the lowest scores on both metrics at 0.8788 and 0.8751, respectively. The exceptional performance exhibited by MLP can be attributed to its capacity to learn complex nonlinear decision boundaries which makes it more effective than traditional machine learning algorithms when dealing with classification problems. Based on these findings, we choose to adopt MLP as it significantly improves the predictive performance of SNMGCDA.

### 3.4 Ablation experiments

In order to further evaluate the generalization and robustness of SNMGCDA, we use ablation experiments to verify the separate contributions of the three feature extraction modules, the experiment by sequentially eliminating individual modules while maintaining the unchanged state of the remaining two modules. In order to guarantee the precision of outcomes, these experiments are performed on identical data samples.

1. Del‐MGA: Remove the multihead graph attention module from SNMGCDA.
2. Del‐SAE: Delete the sparse autoencoder module.
3. Del‐NMF: Remove the NMF module.

**FIGURE 5:**
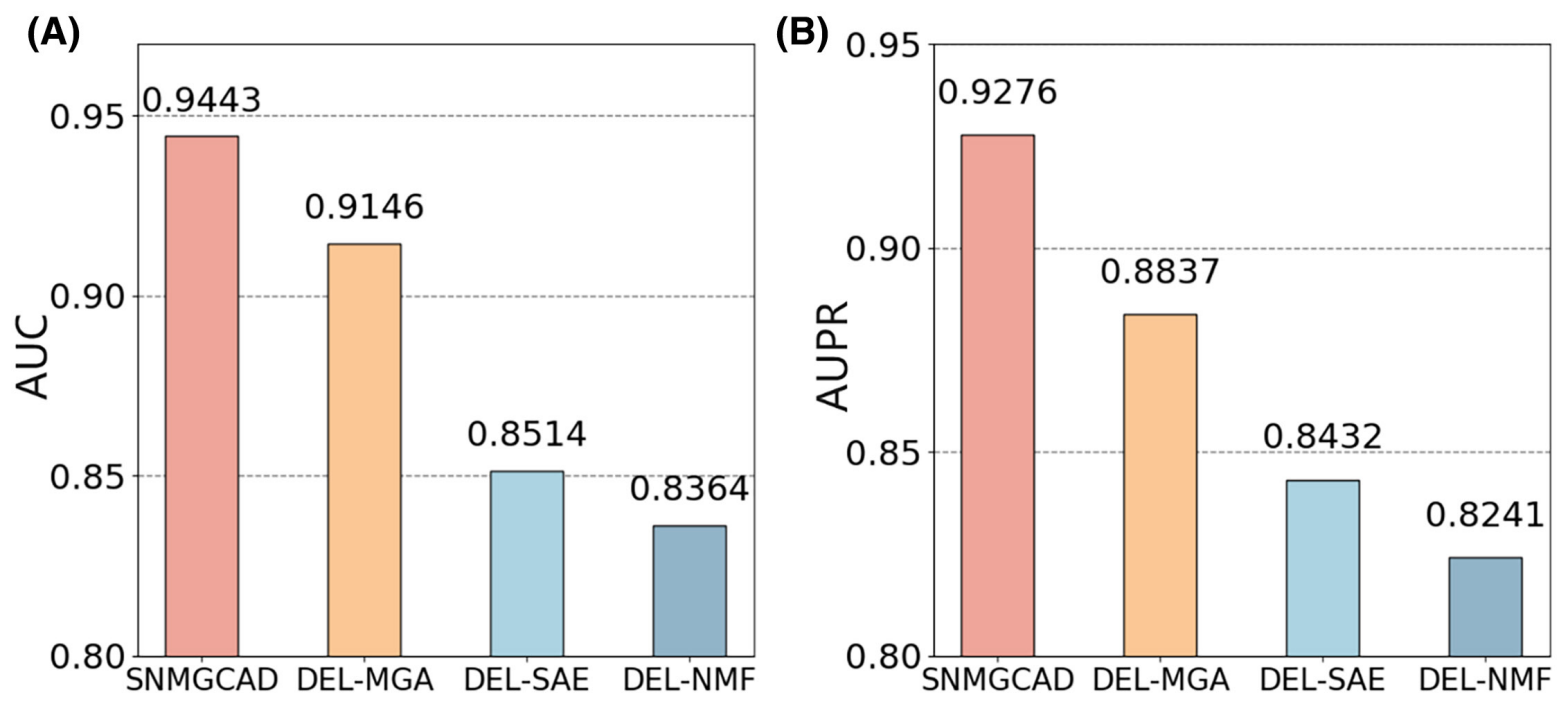
Comparative analysis of the AUC and AUPR values between SNMGCDA and its ablation experiments.

As shown in Figure 5, SNMGCDA shows the highest AUC and AUPR values of 0.9443 and 0.9276, respectively. The AUC and AUPR of Del‐MGA are 0.9164 and 0.8837, respectively. Compared with SNMGCDA, its performance index decreases by 2.79% AUC and 4.39% AUPR, which indicates that the multihead map attention module is significant for our feature training. Then, we remove the sparse autoencoder module and find that all indicators are lower than the results of SNMGCDA, indicating that the sparse autoencoder module plays an important role in feature training. Finally, poor results were obtained when performing Del‐NMF, with AUC and AUPR of 0.8364 and 0.8241, respectively. This emphasizes that 128 drug features and 128 circRNA features extracted by NMF are key components and crucial for SNMGCDA. The reasons why using NMF can lead to better training results may be as follows: NMF can automatically select the most relevant features to decompose, thereby filtering and excluding irrelevant or noisy features, so that the model only focuses on the important features during training, reducing the redundancy and dimensionality of features. The NMF decomposition decomposes the raw data into a combination of topics and weights, which represent some basic patterns or main features in the data. Such a decomposition enables the model to better understand the structure of the data and the relationships between features, thus improving the interpretability of the model. In summary, any of the three modules is indispensable in predicting the potential associations between circRNAs and drugs, and all features play an important role.

### 3.5 Case study

In this subsection, we further verify the accuracy of SNMGCDA prediction results through case studies of two drugs (Piperlongumine and Methotrexate). We utilize the corresponding circRNA‐drug sensitivity relationships from the known database GDSC^37^ as our training dataset and employ an independent database CTRP^47^ for validating new associations predicted by SNMGCDA. The top 20 inference results with highest scores are selected from both drugs for verification.

Piperlongumine, an amide alkaloid derived from Piper plants,^48^ exhibits selective cytotoxicity against various tumour cells, including those of colon, ovarian and liver cancers, while sparing normal cells and avoiding toxic side effects. Moreover, it possesses pharmacological properties such as antimicrobial activity, sedation and anticonvulsant effects.^49^ According to the result presented in Table 4, a total of 17 out of the initial 20 circRNAs predicted to be associated with Piperlongumine have been experimentally validated, and all these confirmed correlations have successfully passed the rigorous Wilcoxon test. The observed false discovery rate (FDR) being less than 0.05 indicates a statistically significant correlation between these circRNAs and Piperlongumine. Additionally, Methotrexate, which is widely recognized as a folic acid antagonist and commonly employed in anticancer chemotherapy,^50^ has demonstrated promising therapeutic potential in preclinical investigations for paediatric tumours, chronic inflammatory skin diseases and other medical conditions.^51^, ^52^ This pharmacological agent holds substantial clinical significance for disease treatment and physiological regulation. As evident from Table 5, validation has been achieved for predictions pertaining to 16 out of the topmost 20 circRNAs associated with Methotrexate within CTRP.

**TABLE 4:** Top 20 circRNAs associated with Piperlongumine.

Ranking	circRNA	Evidence	Ranking	circRNA	Evidence
1	EFEMP1	CTRP	11	KRT19	CTRP
2	MUC16	CTRP	12	PCF11	Nonsignificant
3	ANXA2	CTRP	13	TIMP2	CTRP
4	DBN1	Nonsignificant	14	SPARC	CTRP
5	CALD1	CTRP	15	FAT1	CTRP
6	PSAP	CTRP	16	DDX5	Nonsignificant
7	LTBP3	CTRP	17	MYH9	CTRP
8	FLOT1	CTRP	18	SLC3A2	CTRP
9	KRT19	CTRP	19	PKM	CTRP
10	AHNAK	CTRP	20	ACTN1	CTRP

**TABLE 5:** Top 20 circRNAs associated with Methotrexate.

Ranking	circRNA	Evidence	Ranking	circRNA	Evidence
1	ASPH	CTRP	11	HSP90AB1	CTRP
2	MEF2D	CTRP	12	L1CAM	CTRP
3	SPINT2	CTRP	13	CLSTN1	CTRP
4	KRT19	CTRP	14	KATNB1	Nonsignificant
5	CTSD	CTRP	15	KRT7	CTRP
6	KDELR2	CTRP	16	NGRN	CTRP
7	NES	Nonsignificant	17	CRIM1	CTRP
8	POLR2A	CTRP	18	RAD23B	Nonsignificant
9	MYH9	CTRP	19	KDELR1	CTRP
10	DCBLD2	CTRP	20	CTDSP1	Nonsignificant

We select two distinct drugs, Bortezomib and Nilotinib, which have only one known circRNA‐drug correlation in the dataset, to further assess the predictive capability of the proposed SNMGCDA model. Throughout the training phase, we consider these two drugs' association with circRNAs as unexplored compounds. Bortezomib is a novel proteasome inhibitor with significant sensitization to chemotherapy or radiotherapy, which can overcome tumour resistance when combined with chemotherapy.^53^ Moreover, some clinical research show that bortezomib has a significant improvement in the treatment of haematological malignancies.^54^ Chronic myeloid leukaemia can be effectively treated with Nilotinib, which acts as an inhibitor of Bcr‐Abl tyrosine kinase^55^ and is presently the prevailing therapy for metastatic tumours of the gastrointestinal stroma.^56^ Table 6 shows that seven of the predicted results of the top 10 circRNAs related to bortezomib are consistent with the results in CTRP, and six of the predicted results of the top 10 circRNAs related to nilotinib are consistent with the results in CTRP.

**TABLE 6:** Top 10 predicted circRNAs associated with Bortezomib and Nilotinib.

Bortezomib			Nilotinib		
Ranking	circRNA	Evidence	Ranking	circRNA	Evidence
1	POLR2A	CTRP	1	CTTN	CTRP
2	THBS1	CTRP	2	DDX5	CTRP
3	ANXA2	CTRP	3	ASPH	Nonsignificant
4	PEA15	Nonsignificant	4	GSPT1	CTRP
5	ANP32B	CTRP	5	FAT1	Nonsignificant
6	FBLN1	CTRP	6	MUC16	CTRP
7	TCOF1	Nonsignificant	7	COL1A1	CTRP
8	VIM	CTRP	8	CRIM1	CTRP
9	MEF2D	Nonsignificant	9	PHF14	Nonsignificant
10	CRIM1	CTRP	10	THBS1	Nonsignificant

## 4 DISCUSSION AND CONCLUSION

The rapid development of medical technology has made the association between circRNA and drug sensitivity more and more closely. CircRNA plays a pivotal role in influencing drug sensitivity, thereby facilitating drug discovery and contributing to disease treatment. Therefore, we put forward a deep learning‐based method named SNMGCDA to reveal the potential correlation between circRNA and drug sensitivity. We verify the effectiveness of SNMGCDA by 5‐fold CV and 10‐fold CV on the benchmark datasets, and prove that SNMGCDA has better prediction performance compared with the most advanced methods. Furthermore, independent databases have validated most case studies' results, affirming the effectiveness of SNMGCDA as a tool for foreseeing novel circRNA‐drug sensitivity associations.

The remarkable success achieved by SNMGCDA can be ascribed to a number of pivotal elements. First, we increase the biological information by calculating the structural similarity, Gaussian kernel similarity and information entropy similarity network of drugs, and the sequence similarity, Gaussian kernel similarity and information entropy similarity network of circRNA, and obtain more explanatory prediction results. Second, compared with the traditional linear integration method, the nonlinear integration method is employed for the amalgamation of these similarity networks, which enhances the accuracy of similarity measurement. Third, in terms of circRNAs and drugs, we use three feature extraction modules, which are necessary to achieve outstanding anticipatory outcomes. Finally, we amalgamate all features extracted from each pair of circRNA‐drug to form a more comprehensive and high‐quality feature information vector, and send the feature vector together with the relevant labels to the classifier for training. SNMGCDA impressively shows that the features we extracted are meaningful and of good quality.

Nevertheless, SNMGCDA still demonstrates certain limitations. First, the matrix representing the associations between circRNAs and drugs exhibits a sparse pattern, characterized by a significant disparity in the distribution of positive and negative samples. Second, despite calculating various similarity networks for circRNAs or drugs, the augmented biological information introduces noise and redundancy in features extracted from multiple modules. Last, additional enhancements to specific parameters might be required in order to attain enhanced outcomes. Overcoming these challenges poses a significant task for our future research endeavours. Additionally, there are limitations in specific aspects of the study regarding the following modules. For example, the data used in this study may be limited, and there may be insufficient sample numbers or specific types of circRNAs and drugs not included. This may affect the generalizability of the results. Future studies can further expand the scope of the data set to include more types of circRNAs and drug samples, which will help improve the reliability and generalization performance of the model. In the feature representation aspect, the feature extraction method used in this study may not fully capture the complex features of circRNAs and drugs. Future studies can consider other feature representation methods, such as graph convolutional networks or pretrained deep learning models, to more accurately predict the correlation between circRNAs and drugs sensitivity. In the consideration of other factors, this study mainly focuses on the correlations between circRNAs and drugs sensitivity, but in clinical practice, there may be other factors such as gene mutations and expression levels that may affect drug sensitivity. Future studies can consider incorporating these factors into the model to more comprehensively predict therapeutic responses and drug sensitivity. In summary, existing techniques for computation employ to examine the relationship between circRNA and drug sensitivity are constrained, thus necessitating additional efforts within this research domain.

## AUTHOR CONTRIBUTIONS

**Shuaidong Yin:** Data curation (equal); investigation (equal); methodology (equal); writing – original draft (equal). **Peng Xu:** Data curation (equal); investigation (equal); methodology (equal); writing – original draft (equal). **Yefeng Jiang:** Investigation (equal); validation (equal); visualization (equal). **Xin Yang:** Investigation (equal); validation (equal); visualization (equal). **Ye Lin:** Investigation (equal); validation (equal); visualization (equal). **Manyu Zheng:** Investigation (equal); validation (equal); visualization (equal). **Jinpeng Hu:** Investigation (equal); validation (equal); visualization (equal). **Qi Zhao:** Conceptualization (equal); funding acquisition (equal); investigation (equal); methodology (equal); project administration (equal); resources (equal); writing – review and editing (equal).

## FUNDING INFORMATION

This study was supported by Natural Science Foundation of Liaoning Province (Grant No. 2023‐MS‐288), Fundamental Research Funds for the Liaoning Universities.

## CONFLICT OF INTEREST STATEMENT

The authors declare that the research was conducted in the absence of any commercial or financial relationships that could be construed as a potential conflict of interest.

## Data Availability

The source code and datasets are available online at https://github.com/zhaoqi106/SNMGCDA.
